# Inhibition of Age-Related Cytokines Production by ATGL: A Mechanism Linked to the Anti-Inflammatory Effect of Resveratrol

**DOI:** 10.1155/2014/917698

**Published:** 2014-04-08

**Authors:** Daniele Lettieri Barbato, Giuseppe Tatulli, Katia Aquilano, Maria R. Ciriolo

**Affiliations:** ^1^Department of Biology, University of Rome Tor Vergata, Via della Ricerca Scientifica, 00133 Rome, Italy; ^2^Scientific Institute for Research, Hospitalization and Health Care, Università Telematica San Raffaele, Via di Val Cannuta, 00167 Rome, Italy; ^3^Scientific Institute for Research, Hospitalization and Health Care, San Raffaele Pisana, Via di Val Cannuta, 00163 Rome, Italy

## Abstract

Ageing is characterized by the expansion and the decreased vascularization of visceral adipose tissue (vAT), disruption of metabolic activities, and decline of the function of the immune system, leading to chronic inflammatory states. We previously demonstrated that, in vAT of mice at early state of ageing, adipocytes mount a stress resistance response consisting in the upregulation of ATGL, which is functional in restraining the production of inflammatory cytokines. Here, we found that, in the late phase of ageing, such an adaptive response is impaired. In particular, 24-months-old mice and aged 3T3-L1 adipocytes display affected expression of ATGL and its downstream PPAR**α**-mediated lipid signalling pathway, leading to upregulation of TNF**α** and IL-6 production. We show that the natural polyphenol compound resveratrol (RSV) efficiently suppresses the expression of TNF**α** and IL-6 in an ATGL/PPAR**α** dependent manner. Actually, adipocytes downregulating ATGL do not show a restored PPAR**α** expression and display elevated cytokines production. Overall the results obtained highlight a crucial function of ATGL in inhibiting age-related inflammation and reinforce the idea that RSV could represent a valid natural compound to limit the onset and/or the exacerbation of the age-related inflammatory states.

## 1. Introduction

Immunometabolism is an emerging field of investigation including immunology and biochemical pathways that govern metabolism. Accelerating interest in this area is being fuelled by increased lifespan and the relatively recent knowledge that aging affects the immune system and promotes inflammation that is associated with metabolic dysfunctions [1]. Ageing is associated with an increase in visceral obesity in men and women, which has been claimed as the prominent cause of systemic metabolic perturbations and chronic inflammation [2]. In particular, with ageing, vAT expands and becomes hypovascularized and resident adipocytes quickly release proinflammatory cytokines as response to such stressful condition [3–5]. Among the produced proinflammatory molecules, tumour necrosis factor *α* (TNF*α*) and interleukin 6 (IL-6) have been most intensively studied for their involvement in inducing systemic metabolic perturbations [4].

Polyphenols are the most promising natural compounds to combat metabolic syndromes including age-related inflammatory states [6]. Many polyphenols are efficient antioxidants and anti-inflammatory molecules by virtue of their ability to directly scavenge inflammation-derived radicals, to increase antioxidants expression, and to block inflammatory cytokines production by modulating the activity of specific transcription factors [6]. In adipose tissue, the polyphenol resveratrol (RSV) suppresses both systemic and adipose tissue inflammation and has the potential to improve age-associated metabolic disorders and to increase insulin sensitivity [7, 8]. Moreover, RSV inhibits triglycerides accumulation by suppressing adipocytes differentiation [9] and by stimulating lipid catabolism via the induction of the adipose triglyceride lipase (ATGL) [10].

ATGL is one of the key factors involved in adipose tissue function. It has been firstly identified in white adipose tissue and represents the rate-limiting enzyme of triglycerides lipolysis [11]. Moreover, fatty acids (FAs) liberated by ATGL have been implicated in lipid signalling mediated by the family of peroxisome proliferator activated receptors (PPARs) [12]. PPARs are ligand-activated nuclear receptors, which can be activated by FAs and have been mainly studied in the transcriptional regulation of genes involved in glucose and lipid metabolism [13]. ATGL-mediated FA/PPAR*α* signalling was demonstrated to be essential to maintain mitochondrial oxidative metabolism and in vAT orchestrates stress resistance adaptation of adipocytes to limited nutrient delivery, thus counteracting cell death and the onset of the inflammatory response [5]. ATGL activity changes during aging, and it has been suggested that its expression levels are directly related to the inflammatory status [5, 14, 15]. More precisely, ATGL downregulation is described in several age-related metabolic disorders (i.e., insulin resistance-related states) characterized by an increased level of inflammatory mediators [16, 17]. Importantly, PPARs, including PPAR*α*, play a very important role in the regulation of inflammatory responses. In particular, PPAR*α* transactivates or transrepresses transcription factors including NF*κ*B [18]. However, even though cell metabolism and inflammation are known to be tightly regulated by lipid signalling, the mechanism by which ATGL modulates the production of inflammatory mediators is unclear. In particular, whether FA/PPAR*α* is involved in the anti-inflammatory effect of ATGL has not been fully addressed yet. In the present work we have investigated whether ATGL has a role in orchestrating pro-inflammatory cytokines production in aged adipocytes and whether the anti-inflammatory activity of RSV is associated with modulation of ATGL.

## 2. Materials and Methods

### 2.1. Mice Treatment

We housed and sacrificed all mice in accordance with accepted standard of humane animal care and with the approval by relevant national (Ministry of Welfare) and local (Institutional Animal Care and Use Committee, Tor Vergata University) committees. C57BL/6 male mice were purchased from Harlan Laboratories Srl (Urbino, Italy). For the experiments, 1-, 7-, 14-, and 24-month-old mice were used (*n* = 3 mice/group). Mice were killed by cervical dislocation; vAT was explanted immediately, frozen on dry ice, and stored at −80°C.

### 2.2. Cell Lines, Treatments, and Transfection

3T3-L1 murine preadipocytes and C_2_C_12_ murine myoblasts were purchased from American Type Cell Culture (ATCC) and grown in DMEM supplemented with 10% newborn serum or 10% fetal bovine serum and 1% pen/strep mix (Lonza Sales, Basel, Switzerland). 3T3-L1 and C_2_C_12_ cells were seeded at density of 2 × 10^5^ cells per well in 6-well plates and differentiated in adipocytes and myotubes, respectively, as previously reported [9, 19]. RSV (Sigma-Aldrich, St. Louis, MO, USA) was solubilized in DMSO and added at concentration of 100 *μ*M for up to 48 h, a condition that was demonstrated to be effective in selectively inducing ATGL expression in adipocytes [20]. ATGL and scramble siRNAs (Santa Cruz Biotechnology, Dallas, Texas, USA) were transfected by using the DeliverX Plus kit (Affymetrix, Santa Clara, CA, USA) as previously described [21].

### 2.3. RT-qPCR Analysis

RT-qPCR analysis was carried out as previously described [22]. Briefly, total RNA was extracted using TRI reagent (Sigma-Aldrich). 3 µg of RNA was used for retrotranscription with M-MLV (Promega, Madison, WI, USA). qPCR was performed in triplicates by using validated qPCR primers (BLAST), Ex TAq qPCR Premix (Lonza Sales), and the Real Time PCR LightCycler II (Roche Diagnostics, Indianapolis, IN, USA). mRNA levels were normalized to *β*-actin mRNA, and the relative mRNA levels were determined by using the 2^−ΔΔCt^ method.

### 2.4. Gel Electrophoresis and Western Blotting

Cells and vAT were lysed in RIPA buffer (50 mM Tris HCl pH 8.0, 150 mM NaCl, 0.1% SDS, 0.5% sodium deoxycholate, and 1% NP-40) supplemented with protease inhibitors cocktail (Merck Millipore, Darmstadt, Germany). Western blotting analysis was performed as previously described [23] by using the following polyclonal antibodies: ATGL, *β*-actin, GAPDH, I*κβ*, TNF*α* (Santa Cruz Biotechnologies), and phosphoactive forms of NF*κβ* (pNF*κ*B) (Cell signalling Technologies, Danvers, MA, USA).

### 2.5. Statistical Analysis

The results are presented as means ± S.D. Statistical evaluation was conducted by ANOVA, followed by the post-Student-Newman-Keuls. Differences were considered to be significant at *P* < 0.05.

## 3. Results and Discussion

### 3.1. Age-Related Inflammatory Cytokines Are Produced via an ATGL-Dependent Mechanism in Adipocytes

**Figure 1:**
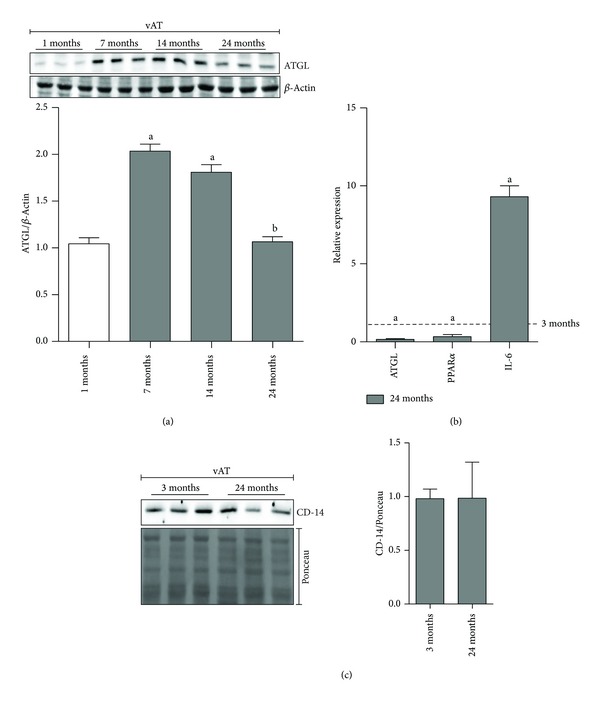
Failure of ATGL/PPAR*α* upregulation is associated with increased IL-6 in vAT of 24-month-old mice. (a)* Upper panel*: western blotting analysis of ATGL in total protein extracts from mice vAT at different age (*n* = 3 mice/group).* Lower panel*: the content of ATGL protein was quantified by densitometric analysis. Data are expressed as ATGL/*β*-actin. Values are given as means ± S.D. ^(a)^ *P* < 0.05 versus 1-month-old mice; ^(b)^ *P* < 0.05 versus 7- and 14-month-old mice. (b) RT-qPCR analysis of relative ATGL, PPAR*α*, and IL-6 mRNA levels in vAT of 3-month- and 24-month-old mice (*n* = 3 mice/group). Values are given as means ± S.D. ^(a)^ *P* < 0.05 versus 3-month-old mice. (c)* Upper panel*: western blotting analysis of CD-14 in total protein extracts from vAT of 3-month- and 24-month-old mice (*n* = 3 mice/group).* Lower panel*: the content of CD-14 protein was quantified by densitometric analysis. Data are expressed as CD-14/Ponceau. *β*-Actin and Ponceau-stained membranes were used as loading controls.

During aging vAT expands and undergoes hypovascularization concomitantly with immunometabolic perturbations [3, 5]. In particular, vAT peaks at middle age or early old age and then declines substantially in advanced old age [24, 25]. The inflammatory state that commonly accompanies aging, also called “inflammaging,” has been proposed to be causative of a systemic metabolic perturbation [26]. vAT has been proposed at the nexus of the mechanisms and pathways involved in the genesis of age-related inflammatory disorders. We have recently demonstrated that during early ageing ATGL is significantly increased in vAT. ATGL upregulation represents a stress adaptive response of adipocytes to hypovascularization that is crucial to buffer energetic catastrophe and to prevent cell death and tissue inflammation [5]. The present study was designed to investigate whether the dramatic inflammatory picture typically observed during late ageing [27] could be triggered by the failure of the ATGL-mediated adaptive response. As showed in Figure 1(a), and in line with what we previously reported, vAT of 7- and 14-month-old mice displayed higher levels of ATGL protein with respect to 1-month-old mice. However, a decline of ATGL was observed at later stage of ageing (Figure 1(a)). In particular, the oldest mice (24-months-old) had ATGL protein level comparable to that of 1-month-old mice (Figure 1(a),* bottom panel*). Moreover, RT-qPCR analysis demonstrated a significant reduction of ATGL mRNA in the oldest mice with respect to young ones, suggesting an impaired ATGL expression (Figure 1(b)). We then attempted to reveal the degree of inflammation in vAT of the oldest mice with respect to the youngest and we found a stronger production of IL-6 mRNA. Notwithstanding, we did not reveal any changes in macrophages marker CD-14 (Figure 1(c)), suggesting that the production of inflammatory cytokines was independent of cellular-mediated immune response. This data is supported by our previous evidence showing that macrophages were not infiltrated in vAT of old mice [5].

**Figure 2:**
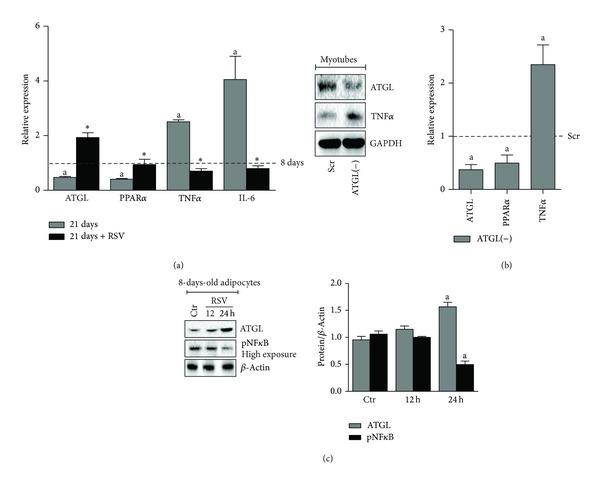
RSV reverts ATGL downregulation and inflammatory markers upregulation in old adipocytes. (a) RT-qPCR analysis of relative ATGL, PPAR*α*, TNF*α*, and IL-6 mRNA levels in 8-day-old (8 d) and 21-day-old (21 d) adipocytes. Cells were treated with 100 *μ*M RSV for 24 h. Values are given as means ± S.D. ^(a)^ *P* < 0.05 versus 8 d; ^(∗)^ *P* < 0.01 versus RSV untreated cells. (b) Differentiated C_2_C_12_ myotubes (4-days-old) were transfected with siRNA against ATGL [ATGL(−)] or with a scramble siRNA (Scr).* Left panel*: western blotting analysis of ATGL, PPAR*α*, and TNF*α* in total protein extracts.* Right panel:* RT-qPCR analysis of relative ATGL and TNF*α* mRNA levels. Values are given as means ± S.D. ^(a)^ *P* < 0.05 versus Scr cells. (c)* Left panel*: western blotting analysis of ATGL and phosphoactive NF*κ*B (pNF*κ*B) in total protein extracts of 8-day-old adipocytes treated with 100 *μ*M RSV for the indicated times.* Right panel*: the content of ATGL or pNF*κ*B protein was quantified by densitometric analysis. Data are expressed as ATGL/*β*-actin or NF*κ*B/*β*-actin. Values are given as means ± S.D. ^(a)^ *P* < 0.05 versus Ctr. GAPDH and *β*-actin were used as loading controls. Data are representative of at least 3 independent experiments.

To confirm the ability of adipose cells residing in vAT of 24-month-old mice to produce inflammatory cytokines independently of cell immunity, we have set up an* in vitro* “aging” model of adipocytes by culturing differentiated 3T3-L1 adipocytes for 21 days and compared the mRNA levels of ATGL, TNF*α*, and IL-6 to those of 3T3-L1 adipocytes after 8 days of differentiation. As shown in Figure 2(a), we detected reduced mRNA level of ATGL and its downstream target PPAR*α*. This event was associated with an upregulation of TNF*α* and IL-6 expression in 21-day-old adipocytes compared with the 8-day-old adipocytes, thus nicely recapitulating the* in vivo* results obtained with 24-month-old mice (Figure 1(b)). Therefore, on the basis of these data we can postulate that the failure of ATGL-mediated stress response observed in 24-month-old and 21-day-old adipocytes triggers the production of proinflammatory cytokines. In agreement with this idea, higher levels of inflammatory markers were observed upon ATGL downregulation* in vitro* and in vAT of ATGL KO mice [5]. The modulatory action of ATGL on tissue inflammation has been reported recently also in cardiac muscle [15]. In particular, a prominent upregulation of different inflammatory markers (e.g., TNF*α* and IL-6) was observed in steatotic hearts of ATGL KO mice. Thus, we asked whether downregulation of ATGL in cultured skeletal muscle myotubes could result in enhanced inflammation markers as well. To this end, we downregulated ATGL in fully differentiated C_2_C_12_ myotubes [ATGL(−)] through RNAi. Coherently, ATGL(−) myotubes displayed decreased PPAR*α* and a greater mRNA expression level of TNF*α* than controls (Figure 2(b)).

FAs are liberated by ATGL function as lipid signalling molecules leading to activation of PPAR*α*, which favours the expression of lipid oxidative genes. Moreover, it has been demonstrated that PPAR*α* functions like a repressor of inflammation [12]. An impaired FA/PPAR*α* signalling was observed during ATGL deficiency in vAT and adipocytes [5] and this may initiate sequelae of events that eventually lead to the induction of proinflammatory genes. In support of this assumption PPAR*α* KO mice show a prolonged inflammatory response [28]. The involved mechanism is that PPAR*α* is a strong repressor of NF*κ*B transcription factor and of its downstream inflammatory cytokines in different cell types including adipocytes [28]. Moreover, PPAR*α* is able to inhibit inflammation in several pathological conditions including hepatic steatosis and obesity [29–31]. The anti-inflammatory role of PPAR*α* in our system is strongly supported by the analysis of PPAR*α* mRNA in 21-day-old adipocytes and in vAT of the 24-month-old mice, which evidenced a significant reduction of PPAR*α* concomitant with production of inflammatory mediators.

### 3.2. RSV Inhibits the Production of Age-Related Proinflammatory Cytokines in Adipocytes by Upregulating ATGL

Several studies have suggested that the health benefits of RSV are mediated by its antioxidant capacity [32]. In the context of adipocytes physiology, RSV strongly inhibits adipogenesis [9] by inducing the synthesis of the main nonenzymatic intracellular antioxidant, that is, glutathione [33]. In doing so, RSV buffers the onset of a prooxidant milieu, which is mandatory for adipocytes differentiation [9]. Other findings support RSV efficacy also in reducing the inflammatory response in several tissues [34] such as brain during neurodegenerative processes [35] and adipose tissue upon high fat and sugar diet [8]. The main anti-inflammatory mechanism of RSV seems to be related to the inhibitory action of NF*κ*B-mediated pathways including the transcription of TNF*α* and IL-6 [36]. According to Lasa et al. [20], we found that treatment of 8-day-old adipocytes with RSV efficiently impinged upon a strong ATGL protein accumulation at 24 h, which was accompanied by the decrease of phosphoactive NF*κ*B (Figure 2(c)). To dissect whether ATGL/FA/PPAR*α* axis could be involved in the anti-inflammaging action of RSV, we analysed the level of ATGL and PPAR*α* after RSV administration in 21-day-old adipocytes. We found a significant upregulation of both ATGL and PPAR*α* expression (Figure 2(a)). Coherently, a simultaneous decrease of TNF*α* and IL-6 mRNA expression was observed after RSV treatment (Figure 2(a)). As reported in the literature, stress stimuli such as LPS or nutrient starvation upregulate ATGL [5, 37]. Moreover, ATGL KO mice challenged with LPS display enhanced inflammation in liver compared to WT and show increased mortality and torpor, and these events have been attributed to impaired PPAR*α* activity [14].

**Figure 3:**
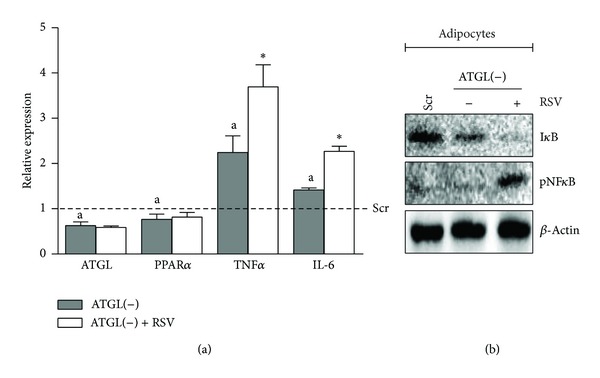
RSV does not revert inflammatory markers upregulation in ATGL lacking cells. (a) Differentiated 3T3-L1 adipocytes (8-days-old) were transfected with siRNA against ATGL [ATGL(−)] or with a scramble siRNA (Scr) and treated with 100 *μ*M RSV for 24 h. RT-qPCR analysis of relative ATGL, PPAR*α*, TNF*α*, and IL-6 mRNA levels. Values are given as means ± S.D. ^(a)^ *P* < 0.05 versus Scr cells; ^(∗)^ *P* < 0.01 versus RSV untreated cells. (b) Western blotting analysis of I*κ*B and phosphoactive form of NF*κ*B (pNF*κ*B) in total protein extracts of adipocytes. *β*-Actin was used as loading control. Immunoblots are representative of at least 3 independent experiments.

Next, given that RSV has an anti-inflammaging action and this nicely correlates with the upregulation of ATGL and PPAR*α*, we asked whether RSV could also restrain the increase of TNF*α* and IL-6 caused by ATGL downregulation through RNAi, in 8-day-old adipocytes. As reported in Figure 3(a), ATGL lacking cells [ATGL(−)] displayed impaired PPAR*α* expression and higher expression of TNF*α* and IL-6 than controls, in line with what we observed in C_2_C_12_ myotubes (Figure 2(b)) and previously revealed on primary adipocytes and mouse embryonic fibroblasts [5]. However, contrary to what we observed in 21-day-old adipocytes, RSV was unable to revert the induction of TNF*α* and IL-6 in ATGL(−) adipocytes; but rather it unexpectedly further upregulated their mRNA expression, indicating an enhancement of NF*κ*B activity (Figure 3(a)). To confirm the proinflammatory role of RSV in ATGL(−) adipocytes, we carried out an immunoblotting analysis of the transcriptional phosphoactive form of NF*κ*B (pNF*κ*B) and its inhibitory partner I*κ*B. Figure 3(b) shows an increased pNF*κ*B level in ATGL(−) adipocytes treated with RSV that was paralleled by the decrease of I*κ*B (Figure 3(b)).

**Figure 4:**
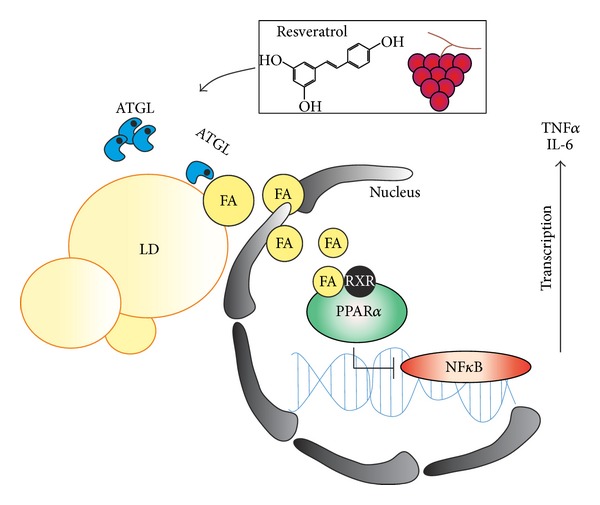
Schematic representation of the ATGL-dependent anti-inflammatory action of resveratrol. Aged adipocytes are characterized by impaired ATGL/FA/PPAR*α* signalling pathways and by the production of proinflammatory cytokines (TNF*α* and IL-6). Resveratrol induces ATGL upregulation, activating FA-mediated PPAR*α* anti-inflammatory effect. ATGL: adipose triglyceride lipase; FA: fatty acid; IL-6: interleukin 6; LD: lipid droplet; NF*κ*B: uclear factor kappa beta; PPAR*α*: peroxisome proliferator activated receptor *α*.

RSV has been proposed as a plausible gerosuppressant natural compound to overcome age-related metabolic perturbations and chronic inflammatory states [8, 38, 39]. Interestingly, in rhesus monkeys, RSV administration reduces NF*κ*B activation in high fat diet fed animals, suppressing inflammation in vAT with beneficial action on metabolic profile [8]. Our data point out that, in condition of irreversible ATGL inhibition (silenced ATGL expression), RSV would not function as anti-inflammatory agent, the ATGL-mediated FA/PPAR*α* signalling axis being strongly affected. Thus, we can speculate that the anti-inflammatory potential of RSV is strongly dependent on ATGL/FA/PPAR*α* pathway (Figure 4).

Overall these findings further support the anti-inflammatory role of ATGL in adipocytes and suggest that RSV, being a powerful enhancer of ATGL expression/activity, is able to reinforce the anti-inflammatory FA/PPAR*α* signalling [5, 15]. Given that RSV is ineffective in inhibiting NF*κ*B when ATGL is lacking, we can suggest that this lipase also efficiently modulates NF*κ*B activity by (i) restraining its phosphorylation and (ii) stabilizing the inhibitory partner I*κ*B. These hypotheses remain to be elucidated yet and are currently under investigation in our laboratory. Importantly, recent papers demonstrate that RSV worsens the clinical symptoms in mice models of multiple sclerosis, exacerbating inflammation and neuronal demyelization [40]. Moreover, RSV has been found to activate NF*κ*B and to increase inflammatory cytokines in cardiac cells [41]. These findings and our data collectively indicate that caution should be exercised in using RSV against inflammatory states.

## 4. Conclusions

Here we give additional effort to the authentic role of ATGL as a stress responsive protein, having the capacity to suppress the production of age-related proinflammatory cytokines in adipocytes. ATGL being an important node in the promotion of lipid signalling, the anti-inflammaging effect of ATGL seems to proceed via the induction of FA/PPAR*α*-mediated pathway in that PPAR*α* functions as a valid suppressor of inflammatory cytokines at the level of gene transcription. Importantly, we have also pointed out that RSV can act as a powerful anti-inflammatory agent thanks to its ability to restore ATGL expression, which is hardly compromised by ageing, thus allowing FA/PPAR*α* signalling to proceed towards the repression of cytokines production. Drugs able to boost activity of ATGL in adipose tissue are not currently available. Therefore, on the basis of our findings we can state that RSV could act as a powerful enhancer of ATGL/FA/PPAR*α* pathway, thus representing a valid natural tool to limit the onset and/or the exacerbation of the age-related metabolic disorders and inflammatory states.

## Abbreviations

ATGL: Adipose triglyceride lipase
FA: Fatty acids
I*κ*B: Nuclear factor of kappa light chain gene enhancer in B-cells inhibitor
IL-6: Interleukin 6
LPS: Lipopolysaccharide
NF*κ*B: Nuclear factor kappa-B
PPARs: Peroxisome proliferator-activated receptors
PPAR*α*: Peroxisome proliferator-activated receptor alpha
RSV: Resveratrol
TNF*α*: Tumour necrosis factor alpha
vAT: Visceral adipose tissue.
